# A High Molecular-Mass *Anoxybacillus* sp. SK3-4 Amylopullulanase: Characterization and Its Relationship in Carbohydrate Utilization

**DOI:** 10.3390/ijms140611302

**Published:** 2013-05-28

**Authors:** Ummirul Mukminin Kahar, Kok-Gan Chan, Madihah Md. Salleh, Siew Mee Hii, Kian Mau Goh

**Affiliations:** 1 Faculty of Biosciences and Medical Engineering (formerly the Faculty of Biosciences and Bioengineering), Universiti Teknologi Malaysia, 81310 Skudai, Johor, Malaysia; E-Mails: ummirulmukminin@gmail.com (U.M.K.); madihah@fbb.utm.my (M.M.S.); grace1832@hotmail.com (S.M.H.); 2 Division of Genetics and Molecular Biology, Institute of Biological Sciences, Faculty of Science, University of Malaya, 50603 Kuala Lumpur, Malaysia; E-Mails: kokgan@um.edu.my

**Keywords:** *Anoxybacillus*, amylase, *Bacillus*, *Geobacillus*, glycoside hydrolase 13, pullulan, pullulanase, starch, thermostable enzyme

## Abstract

An amylopullulanase of the thermophilic *Anoxybacillus* sp. SK3-4 (ApuASK) was purified to homogeneity and characterized. Though amylopullulanases larger than 200 kDa are rare, the molecular mass of purified ApuASK appears to be approximately 225 kDa, on both SDS-PAGE analyses and native-PAGE analyses. ApuASK was stable between pH 6.0 and pH 8.0 and exhibited optimal activity at pH 7.5. The optimal temperature for ApuASK enzyme activity was 60 °C, and it retained 54% of its total activity for 240 min at 65 °C. ApuASK reacts with pullulan, starch, glycogen, and dextrin, yielding glucose, maltose, and maltotriose. Interestingly, most of the previously described amylopullulanases are unable to produce glucose and maltose from these substrates. Thus, ApuASK is a novel, high molecular-mass amylopullulanase able to produce glucose, maltose, and maltotriose from pullulan and starch. Based on whole genome sequencing data, ApuASK appeared to be the largest protein present in *Anoxybacillus* sp. SK3-4. The α-amylase catalytic domain present in all of the amylase superfamily members is present in ApuASK, located between the cyclodextrin (CD)-pullulan-degrading N-terminus and the α-amylase catalytic *C*-terminus (amyC) domains. In addition, the existence of a S-layer homology (SLH) domain indicates that ApuASK might function as a cell-anchoring enzyme and be important for carbohydrate utilization in a streaming hot spring.

## 1. Introduction

Glycoside hydrolases (GHs) are a group of enzymes that catalyze the hydrolysis of glycosidic bonds in carbohydrates. To date, GH families have been divided into 132 groups and reported in the Carbohydrate-Active enZymes (CAZy) database [1]. Most starch-degrading enzymes belong to the GH13 family, which is also known as the α-amylase family [2]. The members within this family share the following, common characteristics: (i) they attack α-glycosidic bonds; (ii) they hydrolyze α-glycosidic bonds to yield α-anomeric, mono- or oligosaccharides (hydrolysis), or form α,1–4 or α,1–6-glycosidic bonds (also known as transglycosylation), or a combination of both activities; (iii) the amino acid sequences contain four conserved regions; and (iv) the enzymes possess a (β/α)_8_ or TIM barrel structure and Asp, Glu, and Asp are the catalytic residues [2]. Often, starch-degrading enzymes possess a carbohydrate-binding module (CBM). The CBMs are currently divided into 67 primary structure-based families [1]. CBMs with affinity for starch (*i.e.*, CBM20, CBM25, and CBM48) are commonly known as starch-binding domains (SBDs) [3]. In general, CBM is a non-catalytic ancillary domain that mediates the attachment of polysaccharide (*i.e.*, starch) granule surfaces to the enzymes and facilitates the degradation process by distorting the conformation and packing of the polysaccharides [3].

Pullulan is a polysaccharide consisting of repeating units of maltotriose joined by α-1,6 glycosidic bonds and a small number of α-1,4 linked maltotetraose units [4]. Pullulan exhibits certain properties desired for dietary and pharmaceutical applications [5]. Pullulanases (pullulan-6-glucanohydrolase) are enzymes that degrade pullulan, starch, and other polysaccharides, yielding various oligosaccharides [6]. Type I and II pullulanases are more frequently reported than other class members. Type I pullulanase (Pul, EC 3.2.1.41) specifically hydrolyzes the α-1,6 glycosidic bonds of pullulan. In contrast, type II pullulanase (amylopullulanase, Apu, EC 3.2.1.1/41) possesses a similar hydrolytic activity to type I but also possesses the ability to hydrolyze α-1,4 glycosidic bonds [4]. In general, Apu enzymes are classified as a member of the GH13 family [2].

The Apu enzyme is employed in conjunction with other amylolytic enzymes (*i.e*., α-amylase and glucoamylase) in industrial starch liquefaction- and saccharification-processing industries because of its catalytic properties [6]. Apu can be used as an additive in laundry and dishwashing detergents [7] and as an antistaling agent in baking [4]. Driven by the demand of the starch-processing industries, considerable efforts have been made to obtain enzymes from thermophiles such as *Geobacillus*, *Thermoanaerobacter*, and *Thermoanaerobacterium* species (Table 1). Several thermostable enzymes from various *Anoxybacillus* species were identified [8], yet an Apu has not been reported. We herein describe the purification and biochemical properties of a high molecular-mass Apu from *Anoxybacillus* sp. SK3-4 (ApuASK). This strain is known for its starch- and pullulan-degrading activities [9]. This study also provides an analysis of the protein sequence and relates the protein sequence with its potential importance in carbohydrate utilization by the cells.

## 2. Results

### 2.1. Genomic Sequencing of Strain SK3-4

The whole genome of *Anoxybacillus* sp. SK3-4 was sequenced using the Illumina MiSeq platform (San Diego, CA, USA). The *de novo* assembly and annotation was performed using the CLC Genomics Workbench 4.8 (CLC Bio, Aarhus, Denmark) and Blast2GO [21] programs. Several glycosyl hydrolase (GH) enzymes that are involved in the degradation of starch or pullulan were data-mined using the dbCAN CAZy web resource [22]. These enzymes include amylopullulanase, ApuASK (C289_2785), α-amylase, ASKA (C289_0468), α-glucosidase (C289_0469), type I pullulanase (C289_2260), glycosidase (C289_2139), and oligo-1,6-glucosidase (C289_0857, C289_1909, and C289_2139). Several putative sugar transporters (C289_0465, C289_0466, C289_0467, C289_0603, C289_0763, C289_0764, C289_0765, C289_0778, C289_0779, C289_0780, C289_1015, C289_1174, C289_1392, C289_1394, C289_1910, C289_1911, and C289_1912) were also found in the Blast2GO annotation. The draft genome was submitted to the National Center for Biotechnology Information (NCBI) Bioproject with accession no. PRJNA174378.

### 2.2. Analysis of the ApuASK Sequence

The *apuASK* gene appeared to be the largest coding open reading frame (ORF) in the entire *Anoxybacillus* sp. SK3-4 genome. The presence of the *apuASK* gene was further confirmed by conventional polymerase chain reaction (PCR) amplification. The intact gene is initiated by a GTG start codon, a 90 bp nucleotide that encodes a signal peptide, and terminates with TAA. The GC content was 43%. The gene consists of 6102 nucleotides that encode a protein containing 2033 amino acid residues (including the 30 amino acid region corresponding to the signal peptide). The theoretical molecular mass of the mature sequence is predicted to be 221,212.6 Da, which is close to the experimentally obtained size (225 kDa). The gene sequence and amino acid sequence for ApuASK are shown in Figure S1.

**Figure 1:**
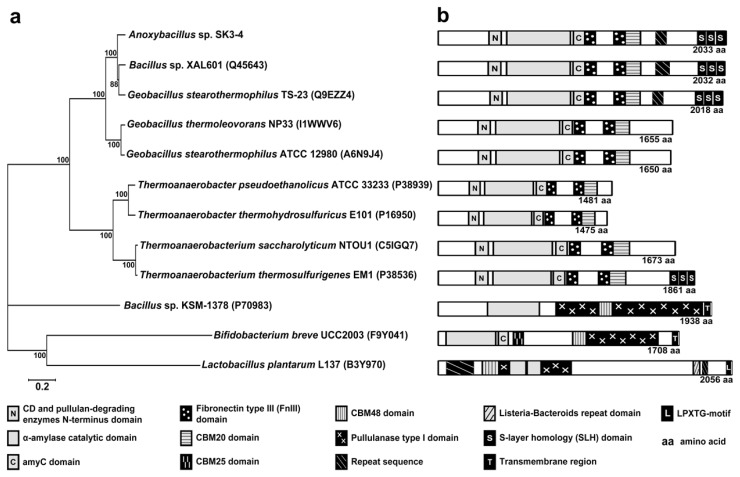
(**a**) The protein relationship tree of Apu from *Anoxybacillus*, *Bacillus*, *Geobacillus*, *Thermoanaerobacter*, *Thermoanaerobacterium*, *Bifidobacterium*, and *Lactobacillus*. (**b**) Schematic representation of conserved domains identified by motif search for the respective Apus.

The relationship between ApuASK and multiple selected Apus is shown in Figure 1a. ApuASK clustered closely with the Apus of *Bacillus* sp. XAL601 (similarity of 90.4%), *Geobacillus stearothermophilus* TS-23 (85.1%), *Geobacillus thermoleovorans* NP33 (74.3%), and *Geobacillus stearothermophilus* ATCC 12980 (71.2%). ApuASK is distinguished from *Thermoanaerobacter* and *Thermoanaerobacterium* Apus, which are only 36.6%–39.2% similar. The distance between ApuASK and the Apus of *Bacillus* sp. KSM-1378, *Bifidobacterium breve* UCC2003, and *Lactobacillus plantarum* L137 are rather long, and the sequence similarity is in the range of only 6%–12%.

ApuASK contains the cyclodextrin (CD) and pullulan-degrading enzyme *N*-terminal domain (A336–T414), the α-amylase catalytic domain (Q452–R912), and the α-amylase catalytic *C*-terminal (amyC) domain (D917–L1001) (Figure 1b). Two fibronectin type III (FnIII) domains (T1006–L1092; Q1214–T1302) are located between the catalytic domains and the CBM20 carbohydrate binding domain (T1302–A1399) (Figure 1b).

The cell-anchoring S-layer homology (SLH) domain (E1811–R1874; F1875–A1936; V1944–M2003) was present in ApuASK. A similar domain is detected in the Apus of *Bacillus* sp. XAL601 and *G. stearothermophilus* TS-23. The Apus of *Bacillus* sp. KSM-1378 [23] and *Bifidobacterium breve* UCC2003 [18] are anchored to the cytoplasmic membrane by a hydrophobic transmembrane structure (Figure 1b). In contrast, the *L. plantarum* L137 Apu is covalently attached to the cell-wall peptidoglycan and associated polymers via the LPXTG-motif [24].

The conserved regions I, II, III, and IV of ApuASK consist of the peptide sequences DGVFNH, GWRLDVANE, EIWD, and LIGSHD, respectively (Figure S1). The WebLogo comparison of these regions among various α-amylases (Amy), cyclodextrin glucanotransferases (CGTase), type I pullulanases (Pul), and amylopullulanases (Apu) is summarized in a supplemental figure (Figure S2).

### 2.3. Purification of Apu

The presence of a signal peptide (Figure S1) indicates that ApuASK is an extracellular enzyme, although a portion of the secreted enzyme could bind to the microbial cell wall via the SLH domain. In a separate experiment, the extracellular, cell-bound, and intracellular fractions of an overnight culture of *Anoxybacillus* sp. SK3-4 were subjected to ApuASK activity. Pullulytic activity was detected in the cell-bound fraction, suggesting that ApuASK is a cell-anchoring enzyme. Relatively higher pullulytic activity was observed in the extracellular fraction (data not shown), which could be due to leaching effects due to over-expressing ApuASK under the experimental conditions.

ApuASK was purified from cell free supernatant using three purification steps that involved ultrafiltration, affinity chromatography, and anion exchange chromatography. ApuASK is a monomeric protein because the purified enzyme had an apparent molecular mass of approximately 225 kDa on sodium dodecyl sulfate-polyacrylamide gel (SDS-PAGE) analyses as well as native-PAGE analyses (Figure S3). A clear band of 225 kDa was also observed on the zymograms prepared to evaluate the pullulytic and amylolytic activities (Figure S3). The zymograms indicated that ApuASK is able to degrade pullulan and starch.

Together with the Apus of *Bacillus* sp. XAL601 (224 kDa) [10], *G. stearothermophilus* TS-23 (220 kDa) [11], *Bacillus* sp. KSM-1378 (210 kDa) [7], and *L. plantarum* L137 (211 kDa) [19], *Anoxybacillus* ApuASK (225 kDa) is among the few Apus that exhibited high molecular-mass (Table 1). In this report, these enzymes are classified as high molecular-mass Apus.

### 2.4. Effects of pH and Temperature on Enzyme Activity and Stability

**Figure 2:**
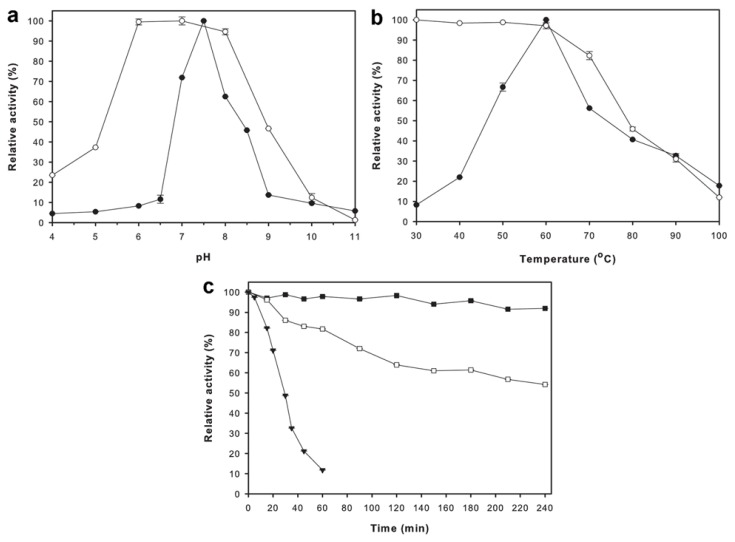
Biochemical characterizations of ApuASK. (**a**) Effects of pH on activity (●) and stability (○) of ApuASK; (**b**) Effects of temperature on activity (●) and stability (○) of ApuASK; (**c**) Thermostability of ApuASK at 60 °C (■), 65 °C (□), and 70 °C (▼). Values are the mean ± standard error of triplicate analyses.

The optimal pH for ApuASK was found to be pH 7.5 (Figure 2a). The enzyme was found to be stable in the pH range of 6.0–8.0, and lost more than 50% of its relative activity at pH 4.0–5.0 and pH 9.0–11.0 (Figure 2a). ApuASK exhibited an optimal temperature at 60 °C (Figure 2b). The enzyme was stable between 30 to 60 °C for 20 min (Figure 2b). The thermostability of ApuASK was further examined over a period of 240 min (4 h) at temperatures ranging from 60 to 70 °C. ApuASK retained more than 90% of its original activity at its optimal temperature (60 °C), whereas ApuASK retained 64% and 54% of its original activity at 65 °C after 120 min and 240 min (4 h) incubation, respectively (Figure 2c).

### 2.5. Effects of Buffers, Metal Ions, and Chemical Reagents

The best buffer for the catalytic activity of ApuASK was potassium phosphate buffer (pH 7.5). In comparison, the enzyme activity in the sodium phosphate and Tris-HCl buffers was reduced by more than 50% (Table 2).

As shown in Table 2, the catalytic activity of ApuASK was enhanced by the addition of K^+^, Fe^2+^, Fe^3+^, Mg^2+^, Mn^2+^, Co^2+^, Cu^2+^, and Ni^2+^. In contrast, the addition of various chemicals reagents significantly affected enzymatic activity. Among the reagents tested, EDTA (chelating agent) strongly inhibited ApuASK with 97% of the original activity lost (Table 2). In the presence of urea (protein denaturant), 75% of the original activity of ApuASK was also lost. Cyclic cyclodextrins (α-, β-, and γ-CDs) reduced the activity to 19, 6, and 2%, respectively, of the original activity (Table 2).

### 2.6. Analysis of the Reaction Products

**Figure 3:**
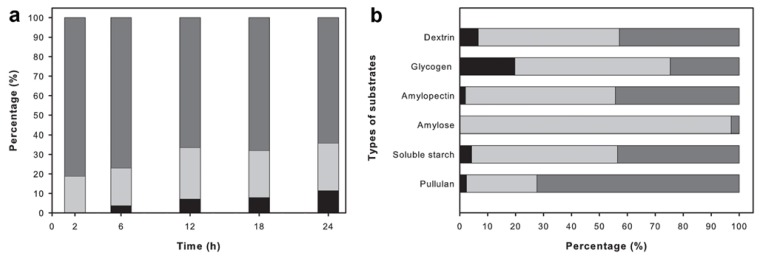
Analysis of reaction products using HPLC. (**a**) Production of glucose (black), maltose (light grey), and maltotriose (grey) by ApuASK on pullulan at different time intervals; (**b**) Production of glucose (black), maltose (light grey), and maltotriose (grey) by ApuASK on individual substrate of pullulan, soluble starch, amylose, amylopectin, glycogen, and dextrin.

The pattern of hydrolysis of ApuASK reacting with pullulan was studied by analyzing the reaction products obtained over the course of 24 h using high performance liquid chromatography (HPLC) (Figure 3a). At the beginning of the time course (2 h), maltotriose was found to be the major sugar type formed and the remaining product was maltose. This suggests that ApuASK preferably cleaves α-1,6 glycosidic bonds rather than α-1,4 glycosidic bonds. In the prolonged reaction (6–24 h), the glucose and maltose fraction continued to increase due to ApuASK degrading the α-1,4 glycosidic bonds (Figure 3a).

Other than pullulan, ApuASK was also able to degrade soluble starch, glycogen, and dextrin to glucose, maltose, and maltotriose, respectively (Figure 3b). The ability of the enzyme to degrade α-1,6 and α-1,4 glycosidic bonds was further determined based on its reaction with amylopectin and amylose, respectively (Figure 3b). ApuASK is classified as an exo-acting enzyme, and these results are in agreement with the description earlier reported [6].

Maltotriose could be mistaken as panose or isopanose because these compounds are eluted from a HPLC column with very similar retention times. To confirm that the hydrolysis product was maltotriose (which possesses only α-1,4 glycosidic bonds) and not panose or isopanose (which possesses both α-1,4 and α-1,6 glycosidic bonds), an established differential approach was used [25]. After ApuASK had reacted with pullulan, the mixture was treated with commercial glucoamylase from *Aspergillus niger* (Sigma-Aldrich, St. Louis, MO, USA) prior to being injected into the HPLC system. Under these conditions, glucose was formed as the sole product (data not shown), indicating that ApuASK produced maltotriose rather than panose or isopanose.

## 3. Discussion

ApuASK exhibited optimal activity at pH 7.5 and 60 °C and stability at marginally acidic and slightly alkaline conditions (pH 6.0–8.0). The pH and temperature tolerances of ApuASK were found to be similar to those of the growth conditions for *Anoxybacillus* sp. SK3-4 [9]. The characteristics of ApuASK are different from those of other high molecular-mass Apus (Table 1). For instance, the Apu of *L. plantarum* L137 is optimally active at pH 4.0 and stable at pH 2.5–6.5 [19], whereas the Apu of *Bacillus* sp. KSM-1378 has an optimal pH of 9.5 and is stable from pH 9.0 to pH 10.0 [7]. The optimal temperature (60 °C) of ApuASK further distinguished itself from those of the other high molecular-mass Apus (Table 1). The enzymes from *L. plantarum* L137 and *Bacillus* sp. KSM-1378 exhibit lower optimal temperatures, of 40 °C [19] and 50 °C [7], respectively. In contrast, the high molecular-mass Apu from *Bacillus* sp. XAL601 has maximal activity at 70 °C [10].

Other high molecular-mass Apus displayed product specificity that is dissimilar from that of ApuASK (Table 1). Analysis of the reaction products revealed that ApuASK possesses an unequivocal ability to only produce glucose, maltose, and maltotriose from pullulan. This characteristic was reported for only one other Apu (100 kDa), the Apu of *Geobacillus* sp. L14 [20].

Analysis of the primary protein sequence revealed that ApuASK and most of the reported Apus have a triad of catalytic domains in a cluster (Figure 1b). First, the CD and pullulan degrading enzyme N-terminus domain functions to assist the binding and hydrolysis of pullulan [26]. The adjacent α-amylase catalytic domain is a region where the hydrolysis of α-1,6 and α-1,4 glycosidic bonds occurs. The third catalytic-related domain, the amyC domain, binds and orientates the α-glucan chains of starch to ensure their proper position so that the enzyme can act upon the starch [27].

ApuASK is a typical Apu with a singular active site for the hydrolysis of α-1,6 and α-1,4 glycosidic bonds. In contrast, the Apus of *Bacillus* sp. KSM-1378 [23] and *Bifidobacterium breve* UCC2003 [18] have an additional type I pullulanase domain (catalytic site) at the C-terminal end (Figure 1b). These two enzymes are known as bi-functional Apus. A bi-functional Apu performs two catalytic activities at two different reaction sites within the same protein [23]. The α-amylase catalytic domain hydrolyzes the α-1,4 glycosidic bonds of the substrates to malto-oligosaccharides while the type I pullulanase domain acts on α-1,6 glycosidic bonds of pullulan, yielding maltotriose and its derivatives [18]. Interestingly, the Apu of *L. plantarum* L137 does not follow the two-domain arrangement of other Apus (Figure 1b).

All of the analyzed Apu enzymes from thermophilic strains of *Anoxybacillus*, *Geobacillus*, *Thermoanaerobacter*, and *Thermoanaerobacterium* species contain CBM20 (Figure 1b). The CBM20 separates the polysaccharide (*i.e.*, starch) chains on the substrate surfaces, hence increasing the accessibility to enzymatic attack [3]. The Apu enzymes of *Bacillus* sp. KSM-1378 and *L. plantarum* L137 employ the CBM48 group instead. Two binding domains, CBM48 and CBM25, were found in the Apu of *Bifidobacterium breve* UCC2003 (Figure 1b). CBM48 functions to facilitate the binding of various polysaccharides particularly glycogen to the enzyme [28] whereas CBM25 assists the binding of α-glucooligosaccharides (particularly containing the α-1,6 glycosidic bonds) and granular starch [29]. In addition, an FnIII domain is found in the Apu enzymes that contain a CBM20 domain but not in Apu enzymes with other types of CBM (Figure 1b). The FnIII domain is composed of a seven-stranded beta sandwich and is found only in extracellular GHases. The domain is non-essential for catalytic reactions, but it may serve as a linker that regulates the binding of the substrate with the enzyme [30].

Nine-repeated sequences of PGSGTT, including interrupts by a PGSGTA and PGSGTM (P1531–T1596), were found in ApuASK (Figures S1 and 1b). An identical stretch of 9 tandem repeats of PGSGTT was found in *G. stearothermophilus* TS-23 Apu [11]. The Apu of *Bacillus* sp. XAL601 has a similar sequence but with a different initial amino acid (GSGTTP) sequence that is replicated 12 times [10]. The repeat regions are most likely folded in a coil at the *C*-terminus; their role is yet to be determined. The repeated sequence (QPT, 50 times) in the Apu of *L. plantarum* L137 is atypical of the aforementioned Apus, and deletion of the repeats did not alter its function [19].

Less is known about the long *C*-terminal region of the Apus in comparison to the knowledge of the catalytic domains in the *N*-terminus. Truncation of the *C*-terminus of the *Bacillus* sp. XAL601 Apu suggested its importance in alkaliphily [31]. The deletion of the *C*-terminal portion of the Apus of *G. stearothermophilus* TS-23 [11] and *G. thermoleovorans* NP33 [12] resulted in contradictory effects on catalytic efficiency. *C*-terminal deletions of the Apus of *Thermoanaerobacterium saccharolyticum* NTOU1 [16] and *Thermoanaerobacter pseudoethanolicus* ATCC 33233 [32] suggested that the *C*-terminus might not be required for enzymatic reaction.

**Figure 4:**
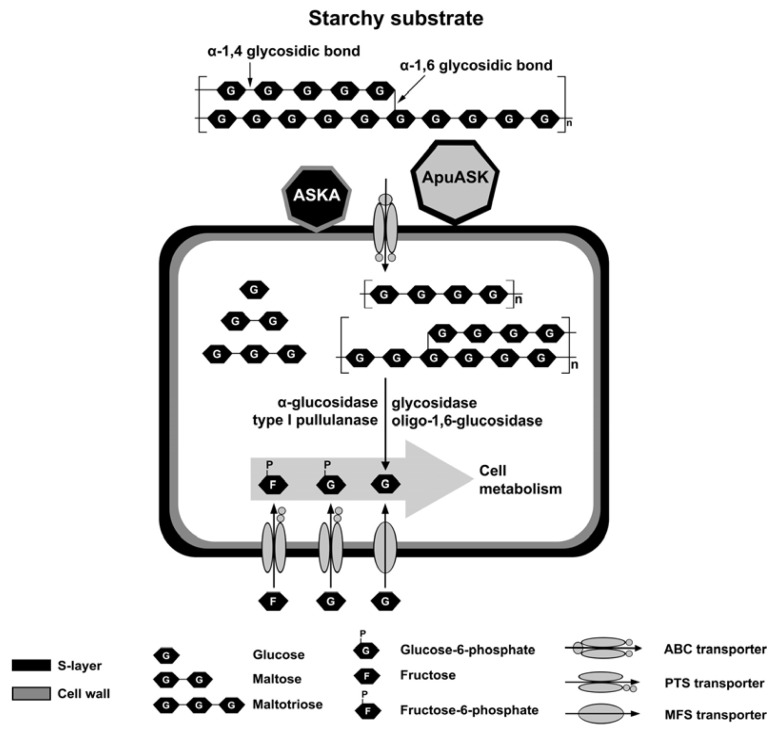
Illustration of carbohydrate (*i.e*., starch) utilization in *Anoxybacillus* sp. SK3-4. Several types of putative transporters and enzymes that are involved in carbohydrate utilization in *Anoxybacillus* sp. SK3-4 were identified through the analyzed sequence data from genome sequencing. The localization of the enzymes was predicted using PSORTb 3.0. ApuASK and α-amylase from *Anoxybacillus* sp. SK3-4, ASKA [33] are cell-bound proteins whiles other enzymes are expressed intracellularly.

Why would the aforementioned bacteria, including *Anoxybacillus* sp. SK3-4, produce high molecular-mass Apus with most of the *C*-terminal regions being unimportant for their catalytic properties? The answer may rest in the cell-anchoring domain near the *C*-terminus. The natural habitat of *Anoxybacillus* sp. SK3-4 is a streaming hot spring where the water flows rapidly. The secreted amylolytic enzymes would be washed away by the stream and be unable to help the cells acquire carbon sources. Cell-bound amylolytic enzymes would be important to degrade starchy substrates. Based on the PSORTb 3.0 predictions for the amylolytic enzymes identified from genome sequencing, ApuASK and α-amylase (designated as ASKA) [33] are the two enzymes that anchor to the cells (Figure 4). Both enzymes hydrolyze starch to glucose, maltose, and maltodextrins that would be transported into the cells via one of the importer systems (Figure 4). Subsequently, these oligosaccharides are converted to glucose through the actions of several intracellular glycosides and a type I pullulanase (Pul). ApuASK and ASKA might be important in the initial stage of starchy substrate degradation.

A gene knockout study demonstrated that Apu plays an important role in carbohydrate metabolism and is essential for the survival of *Bifidobacterium breve* UCC2003 [18]. Although this type of analysis has not been performed with *Anoxybacillus*, the function of its high molecular-mass Apu might be analogous. Experiments to validate this claim using an *Anoxybacillus* knockout approach will be conducted in the near future.

## 4. Experimental Section

### 4.1. Chemicals

All of the chemicals were analytical and molecular grades and purchased from Sigma-Aldrich (St. Louis, MO, USA), unless otherwise stated. Pullulan was obtained from TSI Europe (Zwijndrecht, Belgium). Soluble starch was purchased from the Kanto Chemical Co. Inc. (Tokyo, Japan). Red pullulan was purchased from Megazyme (County Wicklow, Ireland). α-cyclodextrin (α-CD), β-cyclodextrin (β-CD), and γ-cyclodextrin (γ-CD) were purchased from Cyclolab Ltd. (Illatos, Hungary). The freeze-dried epoxy-activated Sepharose 6B medium and the HiTrap Q Fast Flow column were obtained from GE Healthcare (Uppsala, Sweden).

### 4.2. Bacterial Strain, Genome Sequencing, and Protein Sequence Analysis

The *Anoxybacillus* sp. SK3-4 was isolated from the Sungai Klah (SK) hot spring in Malaysia [9]. High-quality genomic DNA was isolated from an overnight culture of *Anoxybacillus* sp. SK3-4 and treated using the protocol suggested by Illumina. Genomic sequencing was performed using the Illumina MiSeq system at the University of Malaya. The draft genome was submitted to the NCBI Bioproject with accession no. PRJNA174378.

The presence of the *apuASK* gene sequence in the genome was validated by PCR amplification using the following primers: forward primer 5′-GTG RRG RGA AGA TGG RRA AAG-3′ and reverse primer 5′-TTA CAT CAA TTT TMC TTT TSY TAA AAA CTC C-3′.

The amino acid sequence of ApuASK and other Apu were analyzed using various programs, including PFAM [34], SMART [35], PROSITE [36], PSORTb 3.0 [37], ClustalW [38], and WebLogo 3.3 [39]. The protein relationship tree was generated using MEGA5 software [40].

### 4.3. Bacterial Culture Conditions

Qualitative screening for pullulytic activity was performed by assessing the formation of clearance zones on *Thermus* medium [9] supplemented with 10 mg/mL of red pullulan. *Anoxybacillus* sp. SK3-4 was routinely cultured in a medium that was optimized for ApuASK production, which contained 7.9 g/L of pullulan, 1.2 g/L of tryptone, 3.9 g/L of ammonium chloride, and 1.0 g/L of MgSO_4_*·*7H_2_O [41]. The initial pH of the medium was 8.29. The culture was incubated at 55 °C with orbital shaking at 200 rpm for 12 h. The cellular localization of ApuASK was determined following the method of Mahajan *et al*. [42].

### 4.4. Determination of Enzyme Activity and Protein Concentration

Apu activity was determined using the 3,5-dinitrosalicylic acid (DNS) method established by Miller [43] with slight modifications. A reaction mixture containing 0.1 mL of enzyme and 1.0 mL of 1.0% (*w*/*v*) pullulan in 100 mM potassium phosphate buffer (pH 7.5) was incubated at 60 °C for 15 min. The DNS reagent (0.5 mL) was then added into the mixture, followed by 50 μL of 0.1 N NaOH. Subsequently, the mixture was boiled for 5 min and the absorbance intensity at 540 nm was measured. Glucose was used as the assay standard. One unit (U) of Apu activity was defined as the amount of enzyme that generated 1 μmol of reducing sugar in 1 min at 60 °C.

The protein concentration was quantified using the Lowry method [44] with bovine serum albumin (BSA) as the standard. The enzyme activity and protein concentration assays were performed at least in triplicate, unless otherwise specified.

### 4.5. Purification of Apu

All of the purification steps were performed at 4 °C unless otherwise specified. The *Anoxybacillus* sp. SK3-4 culture was centrifuged (8000× *g* for 15 min) and the cell-free supernatant was concentrated using a 100 kDa molecular weight cut-off (MWCO) polyethersulfone Vivaflow 50 crossflow ultrafiltration system (Sartorius Stedim Biotech, Aubagne Cedex, France).

The concentrated enzyme was then loaded onto an in-house α-CD epoxy-activated Sepharose 6B column (column volume (CV) of 30 mL). The column was equilibrated with 20 mM sodium phosphate buffer (pH 7.4), and the bound enzyme was eluted with 500 mM NaCl in the same buffer supplemented with 1% (*w*/*v*) α-CD, at a flow rate of 0.5 mL/min. The fractions that had pullulytic activity were pooled and dialyzed overnight against the same buffer in SnakeSkin dialysis tubing with a 10 kDa MWCO (Thermo Fisher Scientific, Rockford, IL, USA).

Subsequently, the dialyzed sample was subjected to a pre-packed HiTrap Q Fast Flow column (CV of 1 mL) equilibrated with 20 mM sodium phosphate buffer (pH 7.4). The bound enzyme was eluted with a linear gradient of 0–100 mM NaCl at a flow rate of 1.0 mL/min. The active fractions were pooled and dialyzed overnight against the same buffer.

### 4.6. Gel Electrophoresis and Zymography

The molecular mass and purity of ApuASK was estimated from electrophoresis in a 12% (*w*/*v*) SDS-PAGE analysis. The enzyme activity (zymography) of ApuASK was evaluated using a 12% (*w*/*v*) native-PAGE analysis. Zymography to determine the pullulytic activity was conducted according to Furegon *et al.* [45], except that the gel was immersed in 100 mM potassium phosphate buffer (pH 7.5) and then incubated at 60 °C for 24 h. Zymography to determine the amylolytic activity was conducted as described by Yang *et al.* [46], except that the starch solution was prepared in 100 mM potassium phosphate buffer (pH 7.5) and the incubation temperature was 60 °C.

### 4.7. Effects of pH and Temperature on Enzyme Activity and Stability

The effect of pH on the activity and stability of ApuASK was determined in a pH range of 4.0 to 11.0. The buffers used were (100 mM of each buffer) the following: sodium acetate (pH 4.0–5.0), potassium phosphate (pH 6.0–7.5), Tris-HCl (pH 8.0–9.0), and glycine-NaOH (pH 10.0–11.0). To determine the pH stability, the enzyme was incubated in the different buffers at room temperature for 30 min without substrate, and then the residual activity was measured under the standard conditions.

The effect of temperature on enzyme activity and stability was determined at temperatures ranging from 30 to 100 °C, at the optimal pH of 7.5. Thermal stability was evaluated by incubating the enzyme without substrate at the different temperatures for 20 min, and then the residual activity was determined using the standard assay conditions. Thermostability was assessed for up to 240 min (4 h); samples were taken at periodic intervals and the residual activity was measured using the standard assay conditions.

### 4.8. Effects of Buffers, Metal Ions, and Chemical Reagents

The influence of the following different buffers on ApuASK activity was investigated (100 mM of each buffer at pH 7.5): potassium phosphate, sodium phosphate, Tris-HCl, MOPS, and HEPES-NaOH. The enzyme was reacted with dissolved substrate in the five different buffers at 60 °C and the relative activity was determined.

The effect of various metal ions and chemical reagents on ApuASK activity was examined. The enzyme was assayed in the presence of 2 mM chloride salts and different concentrations of chemical reagents (Table 2) at 60 °C in 100 mM potassium phosphate buffer (pH 7.5). The residual activity of the enzyme was then determined using the standard assay conditions. The enzyme activity without the additive was the reference (100%).

### 4.9. Analysis of the Reaction Products

A Waters HPLC system with a Waters 2414 refractive index detector (Milford, MA, USA) was used for this analysis. The column employed was a 4.6 × 250 mm 0.5 μm Spherisorb NH_2_ column (Waters, Milford, MA, USA). The internal and external column temperatures were maintained at 30 °C. Acetonitrile:water (70:30, *v*/*v*) was used as the mobile phase, and the flow rate was set at 1.0 mL/min.

The pattern of pullulan hydrolysis by ApuASK was studied for up to 24 h. ApuASK (±1.0 U) was incubated with 2% (*w*/*v*) pullulan in 100 mM potassium phosphate buffer (pH 7.5) at 60 °C. Samples were withdrawn at certain time intervals for 24 h. The enzymatic reaction was then stopped by boiling for 10 min. The insoluble particles were filtered using a 0.45 μm nylon-membrane syringe filter (Whatman, Maidstone, Kent, England). A common differential approach [25] was adopted to determine whether the reaction product of pullulan was maltotriose and not panose or isopanose. The incubation was performed with glucoamylase from *Aspergillus niger* (±1.0 U) in 100 mM sodium acetate buffer (pH 5.0) at 50 °C for 2 h. In general, glucoamylase is capable of degrading α-1,4 glycosidic bonds but not the α-1,6 glycosidic bonds of short-chain oligosaccharides. Glucose, maltose, and maltotriose were used as standards in this analysis. Non-reacted substrates were used as controls.

The ability of ApuASK to hydrolyze six different types of substrates was then studied. ApuASK (±1.0 U) was incubated with 2% (*w*/*v*) of the following substrates: pullulan, soluble starch, amylose, amylopectin, glycogen, and dextrin in 100 mM potassium phosphate buffer (pH 7.5) at 60 °C for 18 h. The enzymatic reaction was then stopped by boiling for 10 min, filtered, and subjected to HPLC under the aforementioned conditions.

### 4.10. Statistical Analysis

The data obtained in this study were analyzed using SYSTAT 12 software (Systat Software Inc., San Jose, CA, USA). The data comparisons with a probability value (*p*-value) of less than 0.05 in the Student’s *t*-test demonstrated that the data were adequate to test all of the hypotheses.

## 5. Conclusions

Knowledge of the high molecular-mass Apus is rather limited. These large enzymes have been found only in *Geobacillus*, *Bacillus*, *Lactobacillus*, and *Thermoanaerobacterium.* This is the first report of a high molecular-mass Apu in *Anoxybacillus*. The ApuASK of *Anoxybacillus* sp. SK3-4 exhibited a high molecular-mass of 225 kDa. ApuASK degrades pullulan, soluble starch, amylose, amylopectin, glycogen, and dextrin to glucose, maltose, and maltotriose. Hence, the unique hydrolytic property of ApuASK, in tandem with its thermostability, suggests that this enzyme could be applied in the starch-processing industry. Currently, all of the other high molecular-mass Apus exhibit product specificity that is different from that of ApuASK. From its biochemical properties and the sequence information obtained from the genome sequencing project, ApuASK appears to be a new high molecular-mass Apu.

## Supplementary Information



## Figures and Tables

**Table 1:** Source and biochemical properties of several known Apus.

Source	TE	MW (kDa)	Opt. temp. (°C)	Opt. pH	Reaction product from pullulan	Ref.
*Anoxybacillus* sp. SK3-4	N	225	60	7.5	Maltotriose, maltose and glucose	This study
*Bacillus* sp. XAL601	R	224	70	9.0	Maltotriose	[10]
*Geobacillus stearothermophilus* TS-23	R	220	ND	ND	ND	[11]
*Geobacillus thermoleovorans* NP33	R	182	60	7.0	Maltotriose	[12]
*Geobacillus stearothermophilus* ATCC 12980	R	184	ND	ND	ND	[13]
*Thermoanaerobacter pseudoethanolicus* ATCC 33233	R	160	ND	ND	ND	[14]
*Thermoanaerobacter thermohydrosulfuricus* E101	R	165	80	ND	Maltotriose	[15]
*Thermoanaerobacterium saccharolyticum* NTOU1	R	100 a	70	5.0	Maltotriose and maltose	[16]
*Thermoanaerobacterium thermosulfurigenes* EM1	R	205	ND	ND	ND	[17]
*Bacillus* sp. KSM-1378	N	210	50	9.5	Maltotriose, maltohexaose and maltononaose	[7]
*Bifidobacterium breve* UCC2003	R	182.3 a	ND	ND	Maltotriose and maltohexaose	[18]
*Lactobacillus plantarum* L137	R	211	40	4.0	Maltotriose	[19]
*Geobacillus* sp. L14	N	100 a	65	5.5	Maltotriose, maltose and glucose	[20]

a approximately; TE = type of enzyme; MW = molecular weight; Opt. temp. = optimum temperature; Opt. pH = optimum pH; Ref. = reference; N = native enzyme; R = recombinant enzyme; ND = not determined.

**Table 2:** Effects of different buffers, metal ions, and chemical reagents on the activity of ApuASK.

Buffers, metal ions, and chemical reagents	Relative activity (%)
Buffers (100 mM, pH 7.5)	
Sodium phosphate	47 ± 0.04
Potassium phosphate	100 ± 0.08
Tris-HCl	45 ± 0.05
MOPS	94 ± 0.02
HEPES-NaOH	15 ± 0.02

Metal ions (2 mM)	
None	100 ± 0.02
Na^+^	91 ± 0.01
K^+^	128 ± 0.01
Fe^2+^	108 ± 0.01
Fe^3+^	196 ± 0.50
Mg^2+^	114 ± 0.02
Mn^2+^	182 ± 0.02
Co^2+^	217 ± 0.03
Cu^2+^	135 ± 0.03
NH_4_^+^	83 ± 0.01
Hg^2+^	16 ± 0.02
Zn^2+^	70 ± 0.02
Ni^2+^	154 ± 0.02
Rb^2+^	14 ± 0.02

Chemical reagents	
None	100 ± 0.02
5 mM EDTA	3 ± 0.04
1 mM SDS	33 ± 0.06
10 mM DTT	45 ± 0.08
10 mM β-mercaptoethanol	27 ± 0.05
3 mM Urea	25 ± 0.06
1% (*v*/*v*) Tween-20	20 ± 0.09
1% (*v*/*v*) Triton X-100	15 ± 0.02
0.1% (*w*/*v*) α-CD	19 ± 0.02
0.1% (*w*/*v*) β-CD	6 ± 0.01
0.1% (*w*/*v*) γ-CD	2 ± 0.01

Values are the mean ± standard error from triplicate analyses.

## References

[1] Cantarel B.L., Coutinho P.M., Rancurel C., Bernard T., Lombard V., Henrissat B. (2009). The carbohydrate-active EnZymes database (CAZy): An expert resource for Glycogenomics. Nucleic Acids Res.

[2] Janeček Š. (2002). How many conserved sequence regions are there in the α-amylase family?. Biologia.

[3] Christiansen C., Abou Hachem M., Janeček Š., Viks⊘-Nielsen A., Blennow A., Svensson B. (2009). The carbohydrate-binding module family 20—diversity, structure, and function. FEBS J..

[4] Domań-Pytka M., Bardowski J. (2004). Pullulan degrading enzymes of bacterial origin. Crit. Rev. Microbiol.

[5] Cheng K.-C., Demirci A., Catchmark J.M. (2011). Pullulan: Biosynthesis, production, and applications. Appl. Microbiol. Biotechnol.

[6] Bertoldo C., Antranikian G. (2002). Starch-hydrolyzing enzymes from thermophilic archaea and bacteria. Curr. Opin. Chem. Biol.

[7] Ara K., Saeki K., Igarashi K., Takaiwa M., Uemura T., Hagihara H., Kawai S., Ito S. (1995). Purification and characterization of an alkaline amylopullulanase with both α-1,4 and α-1,6 hydrolytic activity from alkalophilic *Bacillus* sp. KSM-1378. Biochim. Biophys. Acta.

[8] Goh K.M., Kahar U.M., Chai Y.Y., Chong C.S., Chai K.P., Ranjani V., Illias R.M., Chan K.-G. (2013). Recent discoveries and applications of *Anoxybacillus*. Appl. Microbiol. Biotechnol.

[9] Chai Y.Y., Kahar U.M., Salleh M.M., Illias R.M., Goh K.M. (2012). Isolation and characterization of pullulan-degrading *Anoxybacillus* species isolated from Malaysian hot springs. Environ. Technol.

[10] Lee S.-P., Morikawa M., Takagi M., Imanaka T. (1994). Cloning of the *aapT* gene and characterization of its product, α-amylase-pullulanase (AapT), from thermophilic and alkaliphilic *Bacillus* sp. strain XAL601. Appl. Environ. Microbiol.

[11] Chen J.-T., Chen M.-C., Chen L.-L., Chu W.-S. (2001). Structure and expression of an amylopullulanase gene from *Bacillus stearothermophilus* TS-23. Biotechnol. Appl. Biochem.

[12] Nisha M., Satyanarayana T (2012). Characterization of recombinant amylopullulanase (gt-apu) and truncated amylopullulanase (gt-apuT) of the extreme thermophile *Geobacillus thermoleovorans* NP33 and their action in starch saccharification. Appl. Microbiol. Biotechnol..

[13] Ferner-Ortner-Bleckmann J., Huber-Gries C., Pavkov T., Keller W., Mader C., Ilk N., Sleytr U.B., Egelseer E.M. (2009). The high-molecular-mass amylase (HMMA) of *Geobacillus stearothermophilus* ATCC 12980 interacts with the cell wall components by virtue of three specific binding regions. Mol. Microbiol.

[14] Mathupala S.P., Lowe S.E., Podkovyrov S.M., Zeikus J.G. (1993). Sequencing of the amylopullulanase (*apu*) gene of *Thermoanaerobacter ethanolicus* 39E, and identification of the active site by site-directed mutagenesis. J. Biol. Chem.

[15] Melasniemi H., Paloheimo M. (1989). Cloning and expression of the *Clostridium thermohydrosulfuricum* α-amylase-pullulanase gene in *Escherichia coli*. J. Gen. Microbiol.

[16] Lin F.-P., Ma H.-Y., Lin H.-J., Liu S.-M., Tzou W.-S. (2011). Biochemical characterization of two truncated forms of amylopullulanase from *Thermoanaerobacterium saccharolyticum* NTOU1 to identify its enzymatically active region. Appl. Biochem. Biotechnol.

[17] Matuschek M., Burchhardt G., Sahm K., Bahl H. (1994). Pullulanase of *Thermoanaerobacterium thermosulfurigenes* EM1 (*Clostridium thermosulfurogenes*): Molecular analysis of the gene, composite structure of the enzyme, and a common model for its attachment to the cell surface. J. Bacteriol.

[18] Motherway M.O.C., Fitzgerald G.F., Neirynck S., Ryan S., Steidler L., van Sinderen D. (2008). Characterization of ApuB, an extracellular type II amylopullulanase from *Bifidobacterium breve* UCC2003. Appl. Environ. Microbiol.

[19] Kim J.-H., Sunako M., Ono H., Murooka Y., Fukusaki E., Yamashita M. (2008). Characterization of gene encoding amylopullulanase from plant-originated lactic acid bacterium, *Lactobacillus plantarum* L137. J. Biosci. Bioeng.

[20] Zareian S., Khajeh K., Ranjbar B., Dabirmanesh B., Ghollasi M., Mollania N. (2010). Purification and characterization of a novel amylopullulanase that converts pullulan to glucose, maltose, and maltotriose and starch to glucose and maltose. Enzyme Microb. Technol.

[21] Götz S., García-Gómez J.M., Terol J., Williams T.D., Nagaraj S.H., Nueda M.J., Robles M., Talón M., Dopazo J., Conesa A. (2008). High-throughput functional annotation and data mining with the Blast2GO suite. Nucleic Acids Res.

[22] Yin Y., Mao X., Yang J., Chen X., Mao F., Xu Y. (2012). dbCAN: A web resource for automated carbohydrate-active enzyme annotation. Nucleic Acids Res.

[23] Hatada Y., Igarashi K., Ozaki K., Ara K., Hitomi J., Kobayashi T., Kawai S., Watabe T., Ito S. (1996). Amino acid sequence and molecular structure of an alkaline amylopullulanase from *Bacillus* that hydrolyzes α-1,4 and α-1,6 linkages in polysaccharides at different active sites. J. Biol. Chem.

[24] Kim J.-H., Sunako M., Ono H., Murooka Y., Fukusaki E., Yamashita M. (2009). Characterization of the C-terminal truncated form of amylopullulanase from *Lactobacillus plantarum* L137. J. Biosci. Bioeng.

[25] Rüdiger A., Jorgensen P.L., Antranikian G. (1995). Isolation and characterization of a heat-stable pullulanase from the hyperthermophilic archaeon *Pyrococcus woesei* after cloning and expression of its gene in *Escherichia coli*. Appl. Environ. Microbiol.

[26] Kamitori S., Kondo S., Okuyama K., Yokota T., Shimura Y., Tonozuka T., Sakano Y. (1999). Crystal structure of *Thermoactinomyces vulgaris* R-47 α-amylase II (TVAII) hydrolyzing cyclodextrins and pullulan at 2.6 Å resolution. J. Mol. Biol.

[27] Robert X., Haser R., Gottschalk T.E., Ratajczak F., Driguez H., Svensson B., Aghajari N. (2003). The structure of barley α-amylase isozyme 1 reveals a novel role of domain C in substrate recognition and binding: A pair of sugar tongs. Structure.

[28] Polekhina G., Gupta A., Michell B.J., van Denderen B., Murthy S., Feil S.C., Jennings I.G., Campbell D.J., Witters L.A., Parker M.W. (2003). AMPK β subunit targets metabolic stress sensing to glycogen. Curr. Biol.

[29] Boraston A.B., Healey M., Klassen J., Ficko-Blean E., van Bueren A.L., Law V. (2006). A structural and functional analysis of α-glucan recognition by family 25 and 26 carbohydrate-binding modules reveals a conserved mode of starch recognition. J. Biol. Chem.

[30] Kataeva I.A., Seidel R.D., Shah A., West L.T., Li X.-L., Ljungdahl L.G. (2002). The fibronectin type 3-like repeat from the *Clostridium thermocellum* cellobiohydrolase CbhA promotes hydrolysis of cellulose by modifying its surface. Appl. Environ. Microbiol..

[31] Takagi M., Lee S.-P., Imanaka T. (1996). Diversity in size and alkaliphily of thermostable α-amylase-pullulanases (AapT) produced by recombinant *Escherichia coli*, *Bacillus subtilis* and the wild-type *Bacillus* sp. J. Ferment. Bioeng.

[32] Lin H.-Y., Chuang H.-H., Lin F.-P. (2008). Biochemical characterization of engineered amylopullulanase from *Thermoanaerobacter ethanolicus* 39E-implicating the non-necessity of its 100 *C*-terminal amino acid residues. Extremophiles.

[33] Chai Y.Y., Rahman R.N.Z.R.A., Illias R.M., Goh K.M. (2012). Cloning and characterization of two new thermostable and alkalitolerant α-amylases from the *Anoxybacillus* species that produce high levels of maltose. J. Ind. Microbiol. Biotechnol..

[34] Finn R.D., Mistry J., Tate J., Coggill P., Heger A., Pollington J.E., Gavin O.L., Gunasekaran P., Ceric G., Forslund K. (2010). The Pfam protein families database. Nucleic Acids Res.

[35] Letunic I., Doerks T., Bork P. (2012). SMART 7: Recent updates to the protein domain annotation resource. Nucleic Acids Res.

[36] Sigrist C.J.A., de Castro E., Cerutti L., Cuche B.A., Hulo N., Bridge A., Bougueleret L., Xenarios I. (2013). New and continuing developments at PROSITE. Nucleic Acids Res.

[37] Yu N.Y., Wagner J.R., Laird M.R., Melli G., Rey S., Lo R., Dao P., Cenk Sahinalp S., Ester M., Foster L.J. (2010). PSORTb 3.0: Improved protein subcellular localization prediction with refined localization subcategories and predictive capabilities for all prokaryotes. Bioinformatics.

[38] Larkin M.A., Blackshields G., Brown N.P., Chenna R., McGettigan P.A., McWilliam H., Valentin F., Wallace I.M., Wilm A., Lopez R. (2007). Clustal W and Clustal X version 2.0. Bioinformatics.

[39] Crooks G.E., Hon G., Chandonia J.-M., Brenner S.E. (2004). WebLogo: A sequence logo generator. Genome Res.

[40] Tamura K., Peterson D., Peterson N., Stecher G., Nei M., Kumar S. (2011). MEGA5: Molecular evolutionary genetics analysis using maximum likelihood, evolutionary distance, and maximum parsimony methods. Mol. Biol. Evol.

[41] Kahar U.M., Salleh M.M., Goh K.M. (2013). Medium optimisation for pullulanase production from *Anoxybacillus* species using experimental design. Indian J. Biotechnol..

[42] Mahajan P.M., Desai K.M., Lele S.S. (2012). Production of cell membrane-bound α- and β-glucosidase by *Lactobacillus acidophilus*. Food Bioprocess Technol.

[43] Miller G. L. (1959). Use of dinitrosalicylic acid reagent for determination of reducing sugar. Anal. Chem.

[44] Lowry O.H., Rosebrough N.J., Farr A.L., Randall R.J. (1951). Protein measurement with the Folin phenol reagent. J. Biol. Chem.

[45] Furegon L., Curioni A., Peruffo A.D.B. (1994). Direct detection of pullulanase activity in electrophoretic polyacrylamide gels. Anal. Biochem.

[46] Yang S.-J., Lee H.-S., Park C.-S., Kim Y.-R., Moon T.-W., Park K.-H. (2004). Enzymatic analysis of an amylolytic enzyme from the hyperthermophilic archaeon *Pyrococcus furiosus* reveals its novel catalytic properties as both an α-amylase and a cyclodextrin-hydrolyzing enzyme. Appl. Environ. Microbiol.

