# Supplementary material for: A High Molecular-Mass Anoxybacillus sp. SK3-4 Amylopullulanase: Characterization and Its Relationship in Carbohydrate Utilization
Source: Int J Mol Sci. 2013 May 28;14(6):11302–18. doi: 10.3390/ijms140611302 (PMC3709733; doi:10.3390/ijms140611302)
Supplement: Supplementary file 1 [file ijms-14-11302-s001.pdf]

Supplementary Information

Figure S1. Nucleotide and deduced amino acid sequences of ApuASK. Numbers on left side indicate the positions of nucleotide and amino acid, respectively. Residues from −29 to 0 are signal peptide (underlined). Residues A336−T414 constituted CD and pullulan-degrading enzymes N-terminus domain (○). Residues Q452−R912 indicated alpha-amylase catalytic domain (grey highlight). Residues D917−L1001 represented amyC domain (●). Residues T1006−L1092; Q1214−T1302 constituted two fibronectin type III (FnIII) domains (△). Residues T1302−A1399 indicated CBM20 domain (▲). Residues P1531−T1596 represented nine-repeated sequence of PGSGTT with a PGSGTA and PGSGTM (double underlined). Residues E1811−R1874; F1875−A1936; V1944−M2003 indicated three consecutive repeats of S-layer homology (SLH) domain (★). The four conserved regions of the α-amylase family, designated as I, II, III, and IV are shown in rectangular.

```
                                                                              GTGGGGAGAAGATGG
                                                                               V  G  R  R  W
      AGAAAGCATTTCGCTTATTTCTCAATCTTTTTGCTTTTGGCGCAATTGTTTTCATTTAGTGCAATCGTTCGCGCT
       R  K  H  F  A  Y  F  S  I  F  L  L  L  A  Q  L  F  S  F  S  A  I  V  R  A
   1  AATGAAAACGTGCAAAGCCCTGTCGTAAACGGAAATGAAGTGACGTTCCGTTATGTCGGCACAGGGGAGGAGCAG
   1   N  E  N  V  Q  S  P  V  V  N  G  N  E  V  T  F  R  Y  V  G  T  G  E  E  Q
  76  TCTGTCTTATTAGCCGGATCGTTTAACGGCTGGCAAACAAGTGGAGACAAAAAGATTGAATTGACAAAAGAAAGC
  26   S  V  L  L  A  G  S  F  N  G  W  Q  T  S  G  D  K  K  I  E  L  T  K  E  S
 151  GACCATATTTGGTCAGTCACAAAAACGCTTCCAGACGGAAAGCATATGTACAAGTTTGTTGTGGACGGAAATTGG
  51   D  H  I  W  S  V  T  K  T  L  P  D  G  K  H  M  Y  K  F  V  V  D  G  N  W
 226  AAGCCAGATCCACTAAATAACAATCAAGTAGATGATGGATATGGTGGGAAAAATAGCGTCGTTGTTGTCGGTCAA
  76   K  P  D  P  L  N  N  N  Q  V  D  D  G  Y  G  G  K  N  S  V  V  V  V  G  Q
 301  CCTGTTCAACAACAACGCATCGTCACGCTCGTTGGGAACTTACAAGATGAGTTAGGACATACAGGCGAGTGGGAT
 101   P  V  Q  Q  Q  R  I  V  T  L  V  G  N  L  Q  D  E  L  G  H  T  G  E  W  D
 376  CCAAAAGCGACGGCTACAATTATGAAACATGAAGGCGATGGTTTGTACACGTTTACGGGAACGCTTCCAGCAGGT
 126   P  K  A  T  A  T  I  M  K  H  E  G  D  G  L  Y  T  F  T  G  T  L  P  A  G
 451  ACGTATAAGTACAAAATCGCCATTAACGGTAGTTGGGATGAAAATTACGGTGCTGGTGGACGTGATGGTACAGAT
 151   T  Y  K  Y  K  I  A  I  N  G  S  W  D  E  N  Y  G  A  G  G  R  D  G  T  D
 526  ATTCCACTTACGTTAGAAAAAGAAACGAAAGTGACGTTTTACTATCATGACCGCACGCACGCGATTGCGGACTCG
 176   I  P  L  T  L  E  K  E  T  K  V  T  F  Y  Y  H  D  R  T  H  A  I  A  D  S
 601  ACGTGGTATACACCAATTCCAAAGGAGAAGCAACCGCGTTTAGTCGGAACAATTTTGCCAGCGATCGGTTATGAA
 201   T  W  Y  T  P  I  P  K  E  K  Q  P  R  L  V  G  T  I  L  P  A  I  G  Y  E
 676  ACGGAAGTAAACGGATGGACCCCAGAAAAGTCAACAGCATTTCTCATTGATGATAATTTTGACTCCATTTACACG
 226   T  E  V  N  G  W  T  P  E  K  S  T  A  F  L  I  D  D  N  F  D  S  I  Y  T
 751  TTCACGGCACGTGTGCCAAAAGGGATGTACGAATTTAAGGTGACATTAGGGAATAGCTGGGCCGAAAATTATCCG
 251   F  T  A  R  V  P  K  G  M  Y  E  F  K  V  T  L  G  N  S  W  A  E  N  Y  P
 826  CAAGATGATGCGAAATTAAACGTATTGGAAGATACGACTATAACGTTTTTCTTTAACGATAAAGAAAAAACGTTA
 276   Q  D  D  A  K  L  N  V  L  E  D  T  T  I  T  F  F  F  N  D  K  E  K  T  L
 901  TACACAGACTATAGCCCAACTGGCTCAGATGGGGCAGTGCAAAAGGATCGTTTAAAACATAACACATGGGATTCG
 301   Y  T  D  Y  S  P  T  G  S  D  G  A  V  Q  K  D  R  L  K  H  N  T  W  D  S
 976  TTATATCGCCAACCGTTCGGTGCAGTAAAAGCAGGAGAAAGCGTGACGCTTCGACTAGCGGCGAAAAAAGGGGAT
 326   L  Y  R  Q  P  F  G  A  V  K  A  G  E  S  V  T  L  R  L  A  A  K  K  G  D
                                       ooooooooooooooooooooooooooo

1051  TTAACAAAAGCGAACGTATATGTGAAAAATACAACGACAGGAACAGCGAAGTTATATACAATGAACAAAGTTGGA
 351   L  T  K  A  N  V  Y  V  K  N  T  T  T  G  T  A  K  L  Y  T  M  N  K  V  G
       oooooooooooooooooooooooooooooooooooooooooooo

1126  GTCATCGGTGATGATGAATATTGGGAAGCAACGTTTAAGCCTGATGACAAAGGATTGTACGCATATAAGTTCATT
 376   V  I  G  D  D  E  Y  W  E  A  T  F  K  P  D  D  K  G  L  Y  A  Y  K  F  I
       oooooooooooooooooooooooooooooooooooooooooooo

1201  GCGATGGACGGTTCCGCAAAAGCGGAATATGGAGAGGATACACAAGAAGGTCAATGGGGAAGAGCAGTTGATAAA
 401   A  M  D  G  S  A  K  A  E  Y  G  E  D  T  Q  E  G  Q  W  G  R  A  V  D  K
       oooooooooooooooooooooooo

1276  AATGCTGAATTGTTCCAGCTAACTGTATACGATCCAAACTATAAAACGCCAGATTGGATGAAGCATGCGGTTGTT
 426   N  A  E  L  F  Q  L  T  V  Y  D  P  N  Y  K  T  P  D  W  M  K  H  A  V  V

1351  TATCAAATTTTCCCTGATCGTTTCTTTAACGGCAATCCAGACAATGACGATGCTAAATCGAATGCACGAGGAAAT
 451   Y  Q  I  F  P  D  R  F  F  N  G  N  P  D  N  D  D  A  K  S  N  A  R  G  N
```

```
1426 GAACCAATTGAACATCGCGAATGGTCGCAGCTTCCAGACAATCCACGCATGAAAGGGACAGCTGGATATGATGGC
 476  E  P  I  E  H  R  E  W  S  Q  L  P  D  N  P  R  M  K  G  T  A  G  Y  D  G
1501 GATGGCATTTGGTCGAACGACTTTTTCGGTGGAGATATTGCAGGAATTGAACAAAAGCTAGATTATTTACAATCT
 501  D  G  I  W  S  N  D  F  F  G  G  D  I  A  G  I  E  Q  K  L  D  Y  L  Q  S
1576 CTTGGGGTTAATACGATATATTTAAATCCGATTGCTAAAGCACCATCTAACCATAAATACGATGCGAATGACTAC
 526  L  G  V  N  T  I  Y  L  N  P  I  A  K  A  P  S  N  H  K  Y  D  A  N  D  Y
1651 AAAGAGCTTGATCCAATGTTTGGAACGCCTGAAGAATTTCAATCATTTGTGCAAGCGGTTGCATCTCGTGGTATG
 551  K  E  L  D  P  M  F  G  T  P  E  E  F  Q  S  F  V  Q  A  V  A  S  R  G  M
1726 CACTTAATTTTAGACGGTGTATTTAATCACGTATCAGACGATTCCATTTATTTTGACCGTTATGGAAAATATCCA
 576  H  L  I  L [D  G  V  F  N  H] V  S  D  D  S  I  Y  F  D  R  Y  G  K  Y  P
                  Region I
1801 ACCGTCGGAGCATACGAATATTGGTCGGCTGTGTATGATTTGATGAATGGAAAAGGATTATCAGAAGAACAAGCT
 601  T  V  G  A  Y  E  Y  W  S  A  V  Y  D  L  M  N  G  K  G  L  S  E  E  Q  A
1876 CGTGCTCAAGTTGAACAAAAGTTTAAAGCTGAGGGACAACAGTTCAGTCCGTACGGATTCCATTTATGGTTTAAT
 626  R  A  Q  V  E  Q  K  F  K  A  E  G  Q  Q  F  S  P  Y  G  F  H  L  W  F  N
1951 ATCGAGAATGAAAAAGTAAACGGTGTGTACAAATATCAATCGTGGTGGGGATTTGATAGTTTACCAGAATTTAAG
 651  I  E  N  E  K  V  N  G  V  Y  K  Y  Q  S  W  W  G  F  D  S  L  P  E  F  K
2026 TCTGTCACTGGAACGAAAGTAAAGTATGCGAGCGAGTTAAATAACGAACAGCTTGCAAATTACATTTTTTATGAA
 676  S  V  T  G  T  K  V  K  Y  A  S  E  L  N  N  E  Q  L  A  N  Y  I  F  Y  E
2101 GACGATTCTGTCGCCAAAAGCTGGATTACGCGCGGAGCTTCAGGTTGGCGCTTAGACGTTGCGAATGAAGTCGAT
 701  D  D  S  V  A  K  S  W  I  T  R  G  A  S [G  W  R  L  D  V  A  N  E] V  D
                                                          Region II
2176 ACACAGTTTTGGCGCGAATTTCGTAAAGAGTTGTTGCAAGGTGACTACGACCGTGGCCCAACGTTGAAAAACGGG
 726  T  Q  F  W  R  E  F  R  K  E  L  L  Q  G  D  Y  D  R  G  P  T  L  K  N  G
2251 GAGCAACCGTTAATTCTCGGTGAAATTTGGGATGATGCGTCAAAATACTTCCTAGGCGATCAATACGATTCAGTG
 751  E  Q  P  L  I  L  G [E  I  W  D] D  A  S  K  Y  F  L  G  D  Q  Y  D  S  V
                           Region III
2326 ATGAACTATCGTTTCCGCGGTGCTGTGTTAGACTTTTTACGAAATGGGAAAGCGGAAGATATTGATGCCCGACTA
 776  M  N  Y  R  F  R  G  A  V  L  D  F  L  R  N  G  K  A  E  D  I  D  A  R  L
2401 ACAGCGATTCGTGAAGATTATCCGGAAGAAGCTTTTTATGCGCTAATGAACTTAATTGGTTCACACGATACGGCA
 801  T  A  I  R  E  D  Y  P  E  E  A  F  Y  A  L  M  N [L  I  G  S  H  D] T  A
                                                             Region IV
2476 CGCGCTGTTTTCTTACTTGGAAACGGAACGGATTCATACGAACGTGCTGAACTTGATCCGAATTATAATGAAGAA
 826  R  A  V  F  L  L  G  N  G  T  D  S  Y  E  R  A  E  L  D  P  N  Y  N  E  E
2551 CTCGGTAAAAAGCGATTAAAACTCGCTGCTATTTTACAAATGGGTTATCCAGGTGCGCCGACAATCTACTATGGT
 851  L  G  K  K  R  L  K  L  A  A  I  L  Q  M  G  Y  P  G  A  P  T  I  Y  Y  G
2626 GATGAAGCAGGAGTGACAGGTTCGAAGGATCCGGACGATCGTCGTACGTATCCGTGGGGAAGTGAGGACAAGCAA
 876  D  E  A  G  V  T  G  S  K  D  P  D  D  R  R  T  Y  P  W  G  S  E  D  K  Q
2701 CTCATTGCACATTATCAAAAAGTAGGTGCGATTCGCACGAAGCATCAAGATGTATTAGCGAAAGGAACAATTGAG
 901  L  I  A  H  Y  Q  K  V  G  A  I  R  T  K  H  Q  D  V  L  A  K  G  T  I  E
                                                ●●●●●●●●●●●●●●●●
2776 ACAGTATATGCGAAAGGTGATGTATACGTCTTTGCCCGTCAGTACGGAAAAGATGTTGCGCTTATTGCGGTAAAC
 926  T  V  Y  A  K  G  D  V  Y  V  F  A  R  Q  Y  G  K  D  V  A  L  I  A  V  N
     ●●●●●●●●●●●●●●●●●●●●●●●●●●●●●●●●●●●●●●●●●●
2851 CGCGGCAATAGTGAACAAACGGTACAGTTGGATGTCGCTTCACTTATTCCAAATGGCATCAAATTAGTAGATGAA
 951  R  G  N  S  E  Q  T  V  Q  L  D  V  A  S  L  I  P  N  G  I  K  L  V  D  E
     ●●●●●●●●●●●●●●●●●●●●●●●●●●●●●●●●●●●●●●●●●●
2926 CTACATGATTCATATGATGTAACTGTAACAAATGGAAAAGTCACATTACGTGTTCCAGCAATGGACGGTCGAATG
 976  L  H  D  S  Y  D  V  T  V  T  N  G  K  V  T  L  R  V  P  A  M  D  G  R  M
     ●●●●●●●●●●●●●●●●●●●●●●●●●●●●●●●●●●●●●●●●●●
3001 TTATTTGGAGAAGCAACTGTTGCTCTTCCGAATAAAGTAACAGATGTTCAAGCGACATCGGGCGTTGGACAAGTG
1001  L  F  G  E  A  T  V  A  L  P  N  K  V  T  D  V  Q  A  T  S  G  V  G  Q  V
     ●               △△△△△△△△△△△△△△△△△△△△△△△△△△△△△△△
3076 ACATTAACGTGGGAAGGTGATGCACCAACGTACCGTATTTATCAATCTACATTAAAAGGTGCAGGTTATGAGCTT
1026  T  L  T  W  E  G  D  A  P  T  Y  R  I  Y  Q  S  T  L  K  G  A  G  Y  E  L
     △△△△△△△△△△△△△△△△△△△△△△△△△△△△△△△△△△△△△△△
3151 GTGGAAGAAACGAGTGAAAAGACAGCTGTGATAGACAATTTGAAAAACGGTACCGCATATTACTTTGCGATTACA
1051  V  E  E  T  S  E  K  T  A  V  I  D  N  L  K  N  G  T  A  Y  Y  F  A  I  T
     △△△△△△△△△△△△△△△△△△△△△△△△△△△△△△△△△△△△△△△
3226 GCTGTTGATACAAACGGAAATGAGTCGGCTAAAGTAGAAACAAGCCGCCTTGTCCCACATTATCCATTAGAAGAA
1076  A  V  D  T  N  G  N  E  S  A  K  V  E  T  S  R  L  V  P  H  Y  P  L  E  E
     △△△△△△△△△△△△△△△△△△△△△△△△△△
3301 GAACACGTTGCTTTACTTTCTGAAGTGAACGGTGGAGTGCTCGATTTAGCTGCTTCAATTACGGTTGAAGCGAAA
1101  E  H  V  A  L  L  S  E  V  N  G  G  V  L  D  L  A  A  S  I  T  V  E  A  K
3376 GTGCAAATCGATAATGTAACGAAACATGGGTTAGCTGACGGTTTACAAGCGGTGTTCCAAGTGAAAAAACCGAAT
1126  V  Q  I  D  N  V  T  K  H  G  L  A  D  G  L  Q  A  V  F  Q  V  K  K  P  N
```

```
3451  AGCGACAAATGGGAAGACATACAAGCAACGTACGACCGACAAGATGGCGATGCGAATGTATTCCGTGCATCGTTC
1151   S  D  K  W  E  D  I  Q  A  T  Y  D  R  Q  D  G  D  A  N  V  F  R  A  S  F
3526  ACTCCGCTACAAGCAGGCACGTACACGTATCGTTATGGCTTTACGACAAATCTCGGTGACAGTTGGGTGCATACA
1176   T  P  L  Q  A  G  T  Y  T  Y  R  Y  G  F  T  T  N  L  G  D  S  W  V  H  T
3601  GAAGAAAAAACGTTTACACTCACTGCAAATGAGGCAGATCAACAAGCACCAGCAAAGGGCATTCAACTTGTTCAA
1201   E  E  K  T  F  T  L  T  A  N  E  A  D  Q  Q  A  P  A  K  G  I  Q  L  V  Q
                                             △△△△△△△△△△△△△△△△△△△△

3676  CCAGACGTTGAATCCGGTCAAGTGAATTTAACGTGGTCGTTCGTAGATCGAGATGACAATGATGCCTATATGCTT
1226   P  D  V  E  S  G  Q  V  N  L  T  W  S  F  V  D  R  D  D  N  D  A  Y  M  L
      △△△△△△△△△△△△△△△△△△△△△△△△△△△△△△△△△△△△△

3751  ATCATTGAGCGTGATGGTCAAATCGTCCATACAACAACGAATATCGGTACATCATTTACTGATTATGATGTTGAA
1251   I  I  E  R  D  G  Q  I  V  H  T  T  T  N  I  G  T  S  F  T  D  Y  D  V  E
      △△△△△△△△△△△△△△△△△△△△△△△△△△△△△△△△△△△△△

3826  AACGGAAAAACATATACGTATGTTGTGAAGTTGTATGACCGTGCTGGGAACGTTGTCGCATCAAATGACGTGCAA
1276   N  G  K  T  Y  T  Y  V  V  K  L  Y  D  R  A  G  N  V  V  A  S  N  D  V  Q
      △△△△△△△△△△△△△△△△△△△△△△△△△△△△△△△△△△△△△

3901  ATTACGCCAGACATCGTGATGGTTCAAGTGACGTTTAAAGTGAAAGCACCAAGCTATACACCGTTAGATACGCGC
1301   I  T  P  D  I  V  M  V  Q  V  T  F  K  V  K  A  P  S  Y  T  P  L  D  T  R
      △△▲▲▲▲▲▲▲▲▲▲▲▲▲▲▲▲▲▲▲▲▲▲▲▲▲▲▲▲▲▲▲▲▲▲▲

3976  ATTACGATTCCAAATAGTATTAACGGTTGGAATACAGGTGCATGGGAAATGACGCGTGGTGGAGCGGTCACACCT
1326   I  T  I  P  N  S  I  N  G  W  N  T  G  A  W  E  M  T  R  G  G  A  V  T  P
      ▲▲▲▲▲▲▲▲▲▲▲▲▲▲▲▲▲▲▲▲▲▲▲▲▲▲▲▲▲▲▲▲▲▲▲▲▲

4051  GATTGGGAATTTACAACGGAGCTTCAAGAAGGCGAAACAATTACTTATAAGTATGTAAAGGGTAGCTCATGGGAT
1351   D  W  E  F  T  T  E  L  Q  E  G  E  T  I  T  Y  K  Y  V  K  G  S  S  W  D
      ▲▲▲▲▲▲▲▲▲▲▲▲▲▲▲▲▲▲▲▲▲▲▲▲▲▲▲▲▲▲▲▲▲▲▲▲▲

4126  CAAGAAGGGCTAGCTGATCATACACGTGATGATCAAACAGATGATGATGTTAGCTATTATGGCTATGGTGCGATC
1376   Q  E  G  L  A  D  H  T  R  D  D  Q  T  D  D  D  V  S  Y  Y  G  Y  G  A  I
      ▲▲▲▲▲▲▲▲▲▲▲▲▲▲▲▲▲▲▲▲▲▲▲▲▲▲▲▲▲▲▲▲▲▲▲▲

4201  GGAACGGATTTAAAAGTTACGGTACAAAATCAAGGCAATAACAAAATGATCATTCAAGATTACATTTTACGTTGG
1401   G  T  D  L  K  V  T  V  Q  N  Q  G  N  N  K  M  I  I  Q  D  Y  I  L  R  W
4276  ATCGATATGCCTGTCGTTGTTGAAGAAGTGAAAAAAGATGGCGATCGCGTCACGATAAAAGGAAACGCGATTAAA
1426   I  D  M  P  V  V  V  E  E  V  K  K  D  G  D  R  V  T  I  K  G  N  A  I  K
4351  GATGGCGTGTTAACGATCAATAAAGAACGTGTCACGATTCAAGACGACATGTCATTTACGTACACGTTCACACCA
1451   D  G  V  L  T  I  N  K  E  R  V  T  I  Q  D  D  M  S  F  T  Y  T  F  T  P
4426  GCAGCCAATCAAAAAGAAGTGGCCATCCACATCGAACCGTCTGAACGAAGTAAGTCAGACATCTTTAAAAATGAT
1476   A  A  N  Q  K  E  V  A  I  H  I  E  P  S  E  R  S  K  S  D  I  F  K  N  D
4501  GGCGGTGCCATTGCGAAAAATACGAAAAACTACGTACTAAATATCGAAACAAAACAATTGCGCGAAGGTGTGTTG
1501   G  G  A  I  A  K  N  T  K  N  Y  V  L  N  I  E  T  K  Q  L  R  E  G  V  L
4576  CAAGAGGATACAACACCGGGCAGTGGCACAACACCAGGCAGCGGTACAACACCAGGCAGTGGCACAGCACCAGGT
1526   Q  E  D  T  T  P  G  S  G  T  T  P  G  S  G  T  T  P  G  S  G  T  A  P  G

4651  AGCGGCACAACACCAGGTAGCGGCACAATGCCGGGCAGCGGCACAACACCGGGCAGTGGCACAACACCGGGCAGT
1551   S  G  T  T  P  G  S  G  T  M  P  G  S  G  T  T  P  G  S  G  T  T  P  G  S

4726  GGCACAACACCGGGCAGCGGCACAACACCAGGAAGTGGCACAACACCAGGAAGTGGCACAACACCAGTGAAGGGT
1576   G  T  T  P  G  S  G  T  T  P  G  S  G  T  T  P  G  S  G  T  T  P  V  K  G

4801  GAAAATGGTACGGTTGTTTTACAGCCGAAAGTAGAGACGAAAGAAAAAGACGGCAAAGTAGTAGAAAAAGTGGCA
1601   E  N  G  T  V  V  L  Q  P  K  V  E  T  K  E  K  D  G  K  V  V  E  K  V  A
4876  ACTATTTCAACAAATGAAGTTGAAGCGATTGTCAAGGAGCTGTCGAATGAAAATAAACAAGTCGTCGTCTCCCTC
1626   T  I  S  T  N  E  V  E  A  I  V  K  E  L  S  N  E  N  K  Q  V  V  V  S  L
4951  GGCTCGCTTCCAAAAGGTGTAGCCACAAAAGTAGATGTGCCAGCTACATTATTTACAAAAGCGGCAAATAAGCAA
1651   G  S  L  P  K  G  V  A  T  K  V  D  V  P  A  T  L  F  T  K  A  A  N  K  Q
5026  GGAGAAGCAACGATTGTGAGTGCAACTGAGCAGGCGACATACAAATTGCCAGCAAAAGAAGTACAAGCGTCTCTG
1676   G  E  A  T  I  V  S  A  T  E  Q  A  T  Y  K  L  P  A  K  E  V  Q  A  S  L
5101  GCAGCGATTGCAAAAACACTCGGTGCAACAGTCGAACAAGTAAACATCTCGATCGAAATGAAAGTAAAAGATGCT
1701   A  A  I  A  K  T  L  G  A  T  V  E  Q  V  N  I  S  I  E  M  K  V  K  D  A
5176  CCACAACTACGTGCGAAAGCATTATCTGATGCAGTAGAGTTTCATGTTGTGGCGAAGGCAAACGGAAAAGAACAA
1726   P  Q  L  R  A  K  A  L  S  D  A  V  E  F  H  V  V  A  K  A  N  G  K  E  Q
5251  GTGATTGATCGTTTTACTCAATATGTTGAACGTGAAATCGTATTGAAGCAGGCGGTGAATGCAAGTCGTTCTATT
1751   V  I  D  R  F  T  Q  Y  V  E  R  E  I  V  L  K  Q  A  V  N  A  S  R  S  I
5326  GTGGTGCGCGTAAACGATGACGGTTCCATTACACCTGTACCGACCACATTTGTTGGCAACAAAGCGGTCATCAAG
1776   V  V  R  V  N  D  D  G  S  I  T  P  V  P  T  T  F  V  G  N  K  A  V  I  K
5401  TCATTGACGAACTCGACATACGTCGTCGTTGAAGGAACACATACATTTGGCGACATCGAGCAACATTGGGCGAAA
1801   S  L  T  N  S  T  Y  V  V  V  E  G  T  H  T  F  G  D  I  E  Q  H  W  A  K
                                    ★★★★★★★★★★★★★★★★★★★★★
5476  GGATATATCGAAACATTGGCAGCTAAACAGCTTGTGAAAGGTATGACAGAAACAGCATACCGACCAAATGAGCAG
1826   G  Y  I  E  T  L  A  A  K  Q  L  V  K  G  M  T  E  T  A  Y  R  P  N  E  Q
      ★★★★★★★★★★★★★★★★★★★★★★★★★★★★★★★★★★★★
```

```
5551  ATGACACGTGCCCAGTTTGCTGTGTTGCTTGTACGCGCATTAGCATTGCCACATGAAACATATGACGGTCGATTT
1851   M  T  R  A  Q  F  A  V  L  L  V  R  A  L  A  L  P  H  E  T  Y  D  G  R  F
      ★★★★★★★★★★★★★★★★★★★★★★★★★★★★★★★★★ ★
5626  GCTGATGTAAAAGGCACAGAATGGTTTAACAAAAACGGTGAATTAGCGGCTGCAGTCAAACTAGGTATCATTCAA
1876   A  D  V  K  G  T  E  W  F  N  K  N  G  E  L  A  A  A  V  K  L  G  I  I  Q
      ★★★★★★★★★★★★★★★★★★★★★★★★★★★★★★★★★★★
5701  GGAAAAACAGCCAATACGTTTGCGCCGAATGAGCCAATCACTCGCGCCGAAGCAGCTGTTATGATCGAGCGAGCA
1901   G  K  T  A  N  T  F  A  P  N  E  P  I  T  R  A  E  A  A  V  M  I  E  R  A
      ★★★★★★★★★★★★★★★★★★★★★★★★★★★★★★★★★★★
5776  TTGAAGCTTTCGTTTGTTGGCTATAATGAAGCAACAAACGACAAAACGAAAAAGGTAACAGATTTCCGCGATGCA
1926   L  K  L  S  F  V  G  Y  N  E  A  T  N  D  K  T  K  K  V  T  D  F  R  D  A
      ★★★★★★★★★★★★★★★                          ★★★★★★★★★
5851  AAACAATTGCCAACATGGGCAAAACAGGCGATTGAAGCAGTATACCAAGCAGGAGTCATGCAAGGACGAGATAAT
1951   K  Q  L  P  T  W  A  K  Q  A  I  E  A  V  Y  Q  A  G  V  M  Q  G  R  D  N
      ★★★★★★★★★★★★★★★★★★★★★★★★★★★★★★★★★★★
5926  GGAAGCTTTGATCCTACGAGCCATATGACGCGTGCCGAAATGGCGAAAGTATTGGTGGAGTTTTTAGGAAAAGTA
1976   G  S  F  D  P  T  S  H  M  T  R  A  E  M  A  K  V  L  V  E  F  L  G  K  V
      ★★★★★★★★★★★★★★★★★★★★★★★★★★★★★★★★★★★
6001  AAATTGATGTAA
2001   K  L  M
      ★★★★
```

Figure S2. The comparison of conserved regions of various α-amylase (Amy), cyclodextrin glucanotransferase (CGTase), type I pullulanase (Pul), and amylopullulanase (Apu) using WebLogo 3.3.

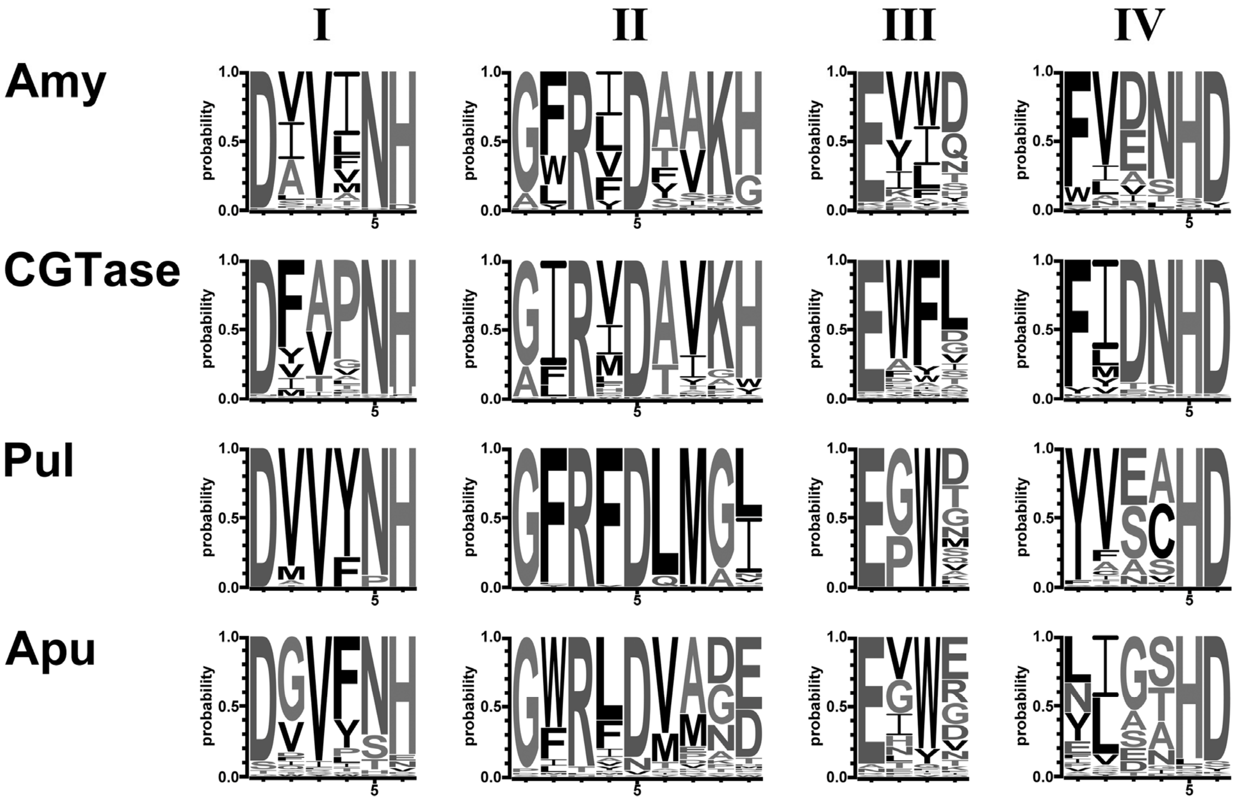

Figure S3. (**a**) SDS-PAGE (12%) and (**b**) Native-PAGE (12%) of the purified ApuASK. Zymogram for (**c**) pullulytic activity and (**d**) amylolytic activity of the purified ApuASK. Lane 1, standard molecular weight marker; Lane 2, purified ApuASK. Arrows pointing the band correspond to the purified ApuASK.

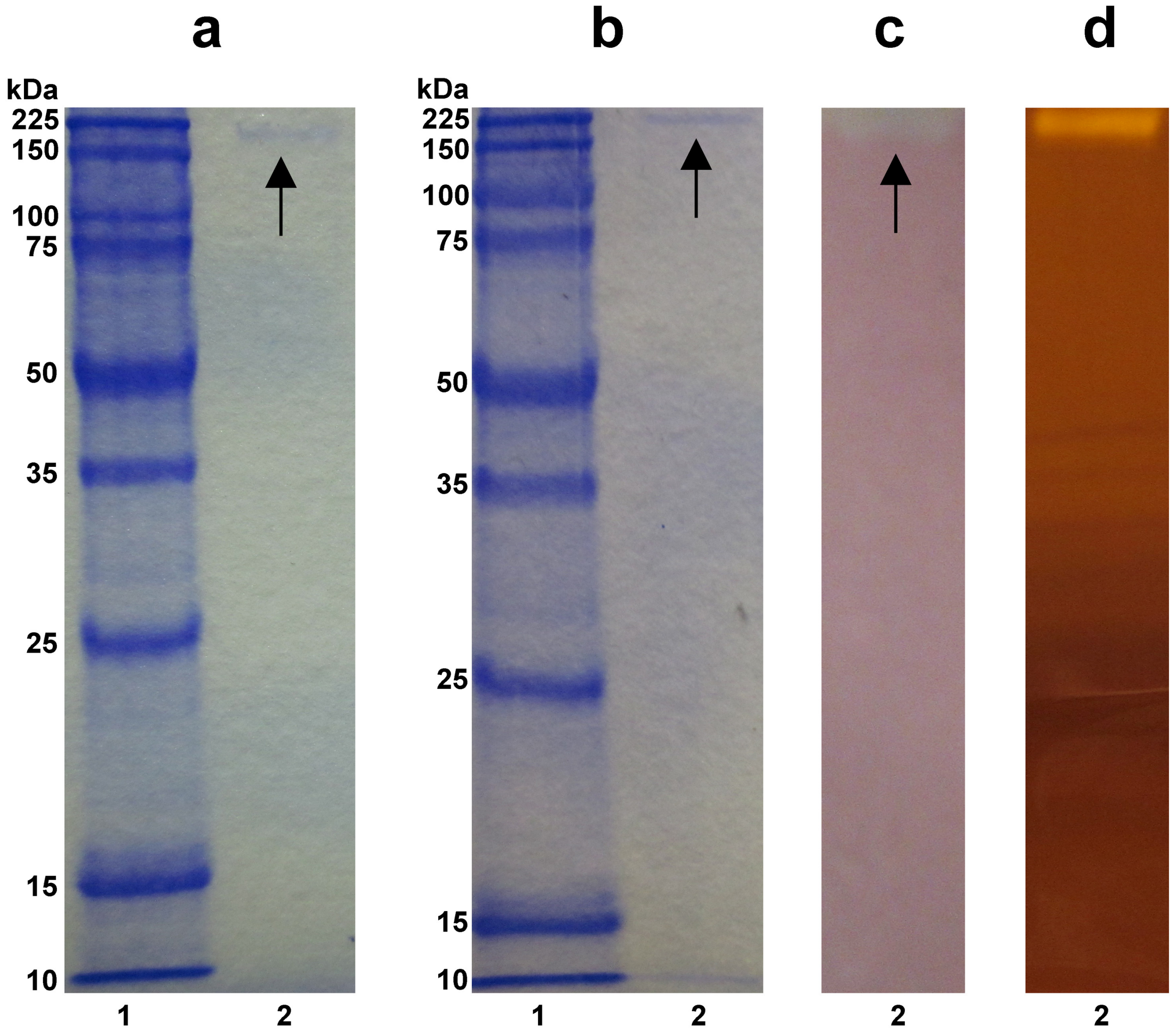

© 2013 by the authors; licensee MDPI, Basel, Switzerland. This article is an open access article distributed under the terms and conditions of the Creative Commons Attribution license (http://creativecommons.org/licenses/by/3.0/).
